# Embolic Transport in LVAD Outflow: An Experimental Study Using Patient-Specific and Idealized Aortic Models

**DOI:** 10.1007/s10439-026-04136-3

**Published:** 2026-04-28

**Authors:** Hamid Mansouri, Muaz Kemerli, Robroy MacIver, Omid Amili

**Affiliations:** 1 Department of Mechanical, Industrial, and Manufacturing Engineering, University of Toledo, Toledo, OH USA; 2 Department of Mechanical Engineering, Sakarya University, Sakarya, Turkey; 3 Children’s Heart Clinic, Children’s Hospitals and Clinics of Minnesota, Minneapolis, MN USA

**Keywords:** LVAD, Patient-specific aortic model, Idealized aortic model, Particle fate, PIV/PTV

## Abstract

A left ventricular assist device (LVAD) is a mechanical pump that provides circulatory support as a bridge-to-cardiac transplantation or as a destination therapy in patients with advanced heart failure. A potential adverse event of LVAD support is thrombus ingestion or formation, which may then travel through the device into the cerebral arteries, causing ischemic strokes. Previous numerical simulations of embolus transport within LVAD systems have exhibited inconsistencies in the results in assessing the fate of emboli in LVAD settings. These disparities prompted the development of an experimental framework tailored for a systematic measurement of particle transport in the context of LVADs. In this *in vitro* study, we utilized a nearly refractive-index-matched time-resolved particle tracking velocimetry (PTV) system to resolve and visualize particle trajectories within each aortic model, complemented by particle image velocimetry (PIV) measurements. We also conducted a meticulous measurement of particle weight in each individual branch by collecting the particles from each outlet. Four LVAD patients, as well as two idealized models of the human aorta, each featuring a cannula grafted at an anastomosis angle of 45 degrees, were considered. Thin-wall high-resolution phantoms of these models were 3D-printed with precision and placed in a flow loop that provided physiological flow conditions. Three different sizes of precision fluorescent beads (neutrally buoyant) with particle-to-cannula diameter ratios of $d_p/D$ = 0.031, 0.053, 0.075 were used to replicate emboli at two clinically relevant flow rates, spanning over 50 experimental cases combined. This systematic investigation reveals that particle distributions largely follow the branchwise flow split, nearly independent of the range of Stokes numbers and inlet Reynolds numbers examined. This finding partially challenges commonly held assumptions in LVAD studies.

## Introduction

Currently, an estimated 6.7 million Americans over the age of 20 are living with heart failure, a number expected to reach 8.5 million by 2030, and the lifetime incidence has been estimated at 24 percent [1], with a global prevalence of approximately 64 million individuals [2]. According to the Heart Failure Epidemiology and Outcomes Statistics report, the prevalence of heart failure will rise to 10.3 million by 2040 and 11.4 million by 2050 [3]. Heart transplantation remains the gold standard therapy for such patients, which provides remarkable improvement in the quality of life and survival. However, it is donor-limited, and this constrained availability of organs contributes to waitlist mortality. Although the number of patients with severe heart failure is increasing significantly [4–6], the number of heart transplants has remained relatively constant [7]. As an alternative, between 2010 and 2021, nearly 30,000 patients in the USA received continuous flow LVAD (CF-LVAD), with over 2500 implants performed annually in recent years [8, 9].

The scarcity of available donor hearts nationwide has increased the demand for mechanical circulatory devices among patients awaiting heart transplants. As the need for heart assist devices grows over time, optimizing these devices and their implantation becomes absolutely necessary. Numerous rapid advancements in the field of ventricular assist device technology have been made to achieve an ideal device that minimizes adverse effects while maintaining good hemodynamic performance [10]. The most recent and widely used addition of the centrifugal-flow device is HeartMate 3 (Abbott Laboratories, Lake Forest, IL), with a fully magnetically levitated rotor and wider blood-flow paths that could significantly reduce shear stress [11]. Although LVADs were initially employed as a bridge to transplant, such advancements in their design have made them increasingly suitable for use as a destination therapy [8, 12, 13].

Despite these advances, the high rotational speeds can damage blood components, which remains a significant challenge and contributes to acute complications. Pump thrombosis and stroke are examples of complications related to LVAD therapy, [11, 14–16] resulting in risks of hemorrhagic/ischemic and thrombotic complications [17–21]. The prior strokes have been reported as one of the significant predictors of death after implant [22, 23]. Despite recent advances in LVAD technology [11, 14, 16, 24], the occurrence of stroke significantly causes morbidity and mortality after LVAD implantation, still remaining problematic [10, 19, 25, 26]. The study of patients implanted with HeartMate 3 by [18] disclosed an 18% rate of stroke incidence in the absence of hemolysis and pump thrombosis, indicating that these events may be linked to other factors. It has been hypothesized that the broader blood flow pathway of HeartMate 3 might unintentionally facilitate the passage of larger thrombi into the systemic circulation, potentially leading to more severe strokes [27–29].

From a fluid dynamics standpoint, the transport of small or large emboli to the aortic arch is fundamentally a fluid-particle interaction problem. The intuitive opinion is that particles generally favor a distribution proportional to the fluid flow division. However, several studies pointed out the surprising predilections regarding the fates of particles. Previous investigations of particle transport through the idealized Y-shaped bifurcation, such as [30] and [31], demonstrated that suspended particles tend to preferentially enter the wider diameter branches beyond the distribution of the flow division, even though the particles were small enough to pass through either branch. An *in vitro* study of emboli transport in the cerebral artery by [32] revealed that the larger diameter emboli favored entering the wider diameter artery (middle cerebral artery) than the smaller diameter artery (anterior cerebral artery). A computational modeling of particle transport from aortic arches to the cerebral arteries demonstrated that particle fate is different from volumetric flow division [33]. [34] examined how the graft angle, using the descending aorta as the graft site, and different pulse modulation patterns influence particle distribution and hemodynamic parameters. Their findings indicated that particle dispersion is strongly dependent on these configuration settings.

Several studies (mostly numerical simulations) investigating the impact of cannula grafting angles on trajectories of embolic particles in LVAD systems have yielded mixed results. The computational fluid dynamics (CFD) study by [35] and [36], considering three particle sizes (2, 4, and 5 mm) and three different cannula implementation angles 0, 30, and 60 degrees showed that the shallower angle (60 degrees) prevented the formation of recirculation zones in the ascending aorta. The angles are the orientation of the cannula relative to the surface of the ascending aorta at the insertion site; i.e., 60 degrees is the shallower angle with respect to the ascending centerline. They also showed that particle transportation toward the cerebral arteries is significantly reduced by a shallower angle. [37] leveraged a computational approach and found that the 30-degree model is the best angle under unsteady flow, whereas the 60-degree model (shallower angle) exhibits better results when the flow was presumed steady. A CFD study by [38] considering 3 μm particle size, proposed that the two shallower angles among (30, 60, and 90 degrees with respect to the vertical centerline of the ascending aorta) were least thrombogenic, whereas the 90 degree configuration was most thrombogenic. [39, 40] solved the particle equation of motion (for spheres in the range of 0.1−1.0 mm) using MRI-reconstructed flow fields to identify the optimal cannula outflow configurations. They found that the 90-degree angle was the most favorable among several realistic configurations. An unladen flow study by [41] revealed that a larger anastomosis angle yields the highest flow rate and smallest retrograde flow in the ascending aorta, better supporting heart contractility compared to a smaller angle.

As such, we observe that the angle and position of the cannula graft strongly affect thrombotic events. However, the optimal grafting angle for a cannula is not consistent across different metrics and methods, producing varying results on which angle most effectively reduces the incidence of strokes [35–38, 40, 41]. These inconsistencies in prior numerical simulations, along with the scarcity of supporting experimental validations, motivated us to develop a robust imaging setup for a systematic study of particle dynamics in LVAD settings. The present work experimentally examines embolic transport in four LVAD patient-specific anatomies, each grafted with a similar cannula anastomosis angle of approximately 45 degrees. Additionally, two idealized aortic models recently developed by the authors [42] are used to further extend the applicability of our patient-specific results to the general population.

For each aortic model, we carried out a meticulous set of particle weight measurements using fluorescent particles of different sizes to quantify branch-specific particle ratios and compare particle deposition patterns with the baseline flow split. Additionally, through particle imaging, their fates are evaluated and color-coded based on their branch destinations. We present a selection of inertial particle trajectories using particle tracking velocimetry (PTV), as well as unladen flow velocity fields measured using particle image velocimetry (PIV). Notably, particle weight measurements were obtained for all models, while imaging was performed only for the idealized models for practical purposes. All measurements were conducted at two steady flow rates, corresponding to common and peak cardiac output.

## Materials and Methods

**Fig. 1:**
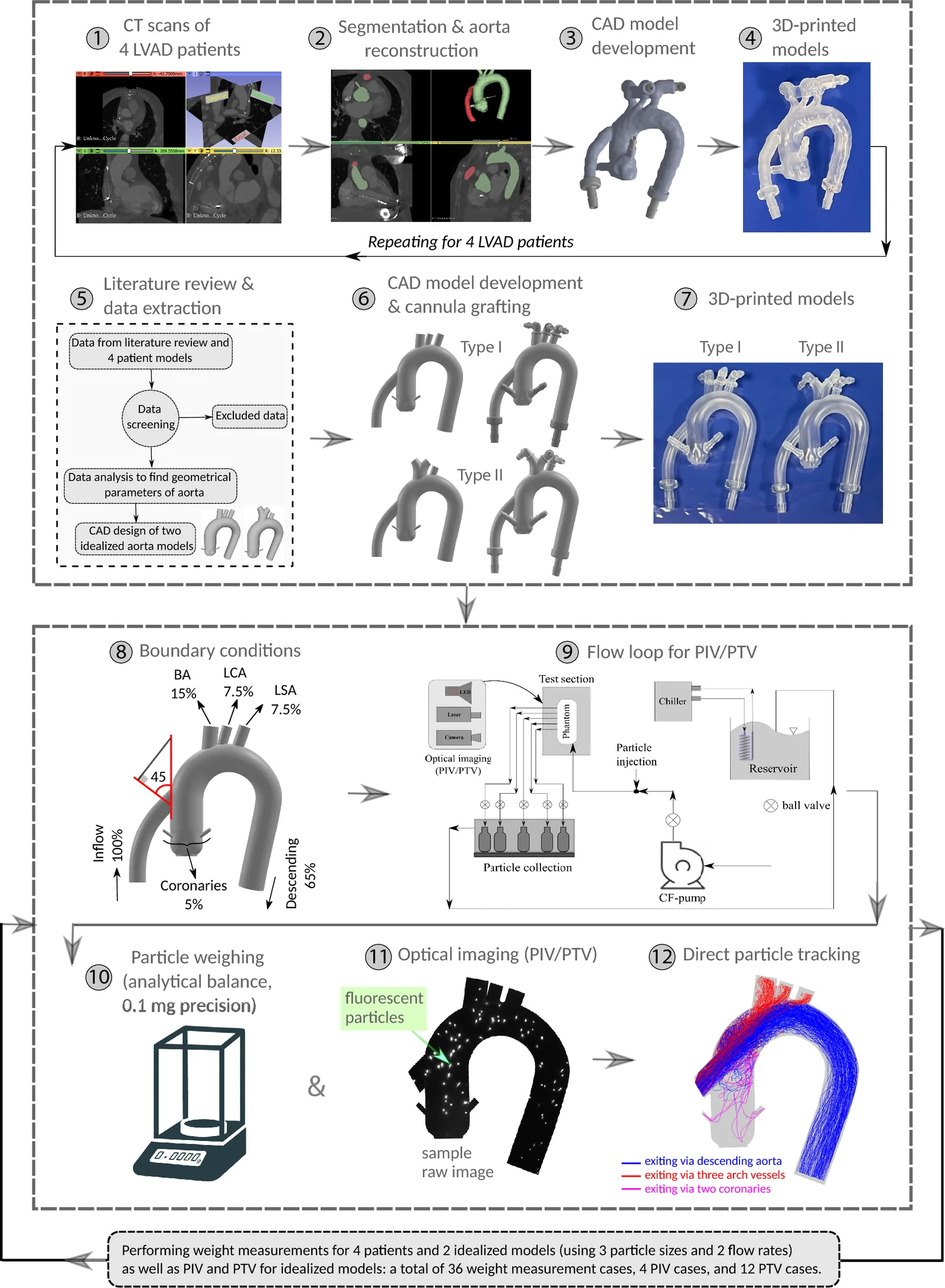
Workflow summarizing the major steps of the study

This section illustrates the study’s workflow, depicted in Fig. 1, which visually outlines the key steps. These steps include the sequential preparation of patient-specific and idealized models, alongside experimental measurements. The study relies on two methodologies: particle weight assessment and PIV/PTV experiments. Measurement uncertainties related to weight measurements, their convergence, and image distortion arising from optical imaging are also elaborated.

### Patient-Specific Models

The high-resolution CT scans of four de-identified LVAD patients with an average age of 72.75 ± 9.32 years were provided to the authors. The requirement of ethical approval was waived by Allina Health IRB 1 (Reference Number: 1542852-1) and The University of Toledo (Reference Number: 300885) for the studies involving humans because the institutional review boards determined that this study does not meet the definition of research or research with human subjects.

Segmentation was performed using 3D Slicer^®^ software, an open-source platform sponsored by the National Institutes of Health (NIH). The angle of the native grafted cannula was found to be approximately 45 degrees across all cases. Steps 1 to 4, as illustrated in Fig. 1, outline the preparation process of the patient’s models. Step 1 involves uploading CT images to the software, which are typically processed through three different views, including the sagittal, coronal, and axial perspectives. Step 2 demonstrates a semi-manual segmentation process to extract the aorta’s 3D geometry. In step 3, a shell with a wall thickness of 1.0 mm is created, then tube barbs are added digitally using SolidWorks in order to connect models to the inflow and outflow tubes. The final digital model, as shown in step 4, is then 3D printed.

**Fig. 2:**
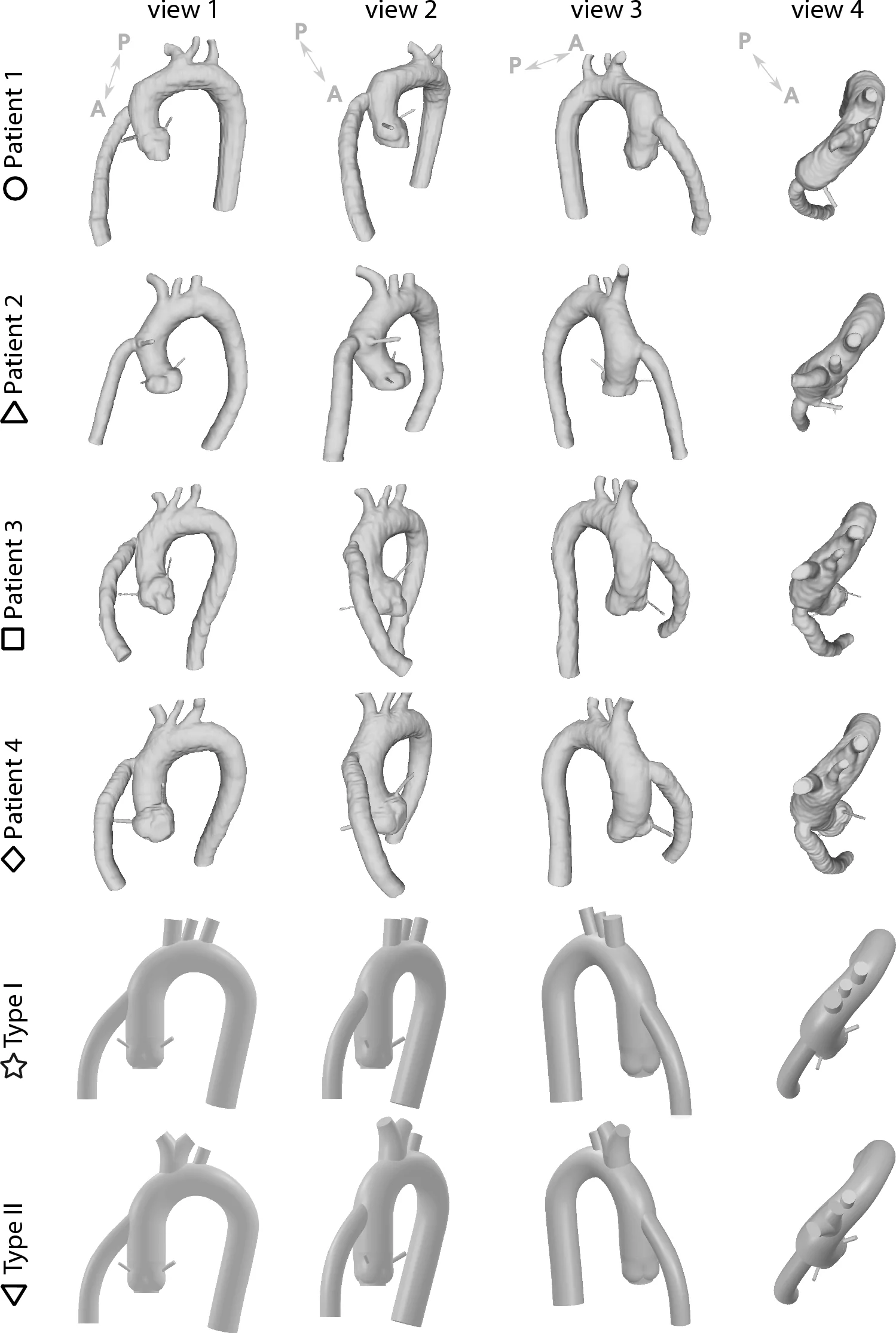
Four reconstructed lumen views from CT scans of LVAD patients, alongside two idealized aortic models. The native grafted cannula for each patient is approximately 16 mm in diameter with a graft angle of approximately 45 degrees. A cannula with the same diameter and contact angle was grafted digitally to idealized aortic models. The arrows represent the anterior (A) and posterior (P) directions. Each model is labeled with a unique marker shape, which is used consistently throughout the study

### Idealized Aortic Models

Following a meticulous assessment of healthy adult aortic morphology and dimensions, we developed two computer-aided design (CAD) models. The details about these aorta models (without a cannula graft) are presented by the authors in [42]. Steps 5, 6, 7 in Fig. 1 demonstrate the design process of idealized aortic models. Step 5 shows a literature review, data extraction, and data analysis for the model development. Step 6 displays the 3D CAD model development in SolidWorks, where a shell with a wall thickness of 0.6 mm is created, and the cannula and tube barbs are added.

In [42], key aortic morphological parameters and branching patterns were compiled from a large number of image-based and cadaveric studies using cohort-size-weighted averages, supplemented by CT data from four recruited subjects. The resulting idealized geometries represent (i) the normal arch with three distinct branches (brachiocephalic artery, BA, left common carotid artery, LCA, and left subclavian artery, LSA), reported in ∼80% of the population, and (ii) the bovine arch, in which the BA and LCA originate from a common trunk (while the LSA branches directly from the aortic arch), reported in ∼14% of individuals. Collectively, these two idealized models namely the Normal variant (Type I) and the Bovine variant (Type II) are estimated to capture the major anatomic features of the healthy adult human aorta for approximately 94% of the general population. Detailed information on the development of the idealized models can be found in [42]. To conduct experiments with these two idealized models, we digitally added a cannula of an assist device into the ascending aorta at a clinically common location and angle, matching the grafting angle (∼45 degrees) and inner diameter (16 mm) observed in our patient-specific cases. The final digital models are then 3D printed in step 7. Figure 2 illustrates different views of four patient-specific and two idealized aortic models. The arrows represent the posterior and anterior views of the models, labeled as ’P’ and ’A’, respectively.

### Model’s Fabrication

The rigid models of patients and idealized cases were manufactured by 3D printing (scale 1:1) at the W. M. Keck Center, University of Texas at El Paso. High-resolution stereolithography (SLA) of Somos^®^WaterShed XC11122 resin at 100 μm layers for patient models (with a wall thickness of 1.0 mm) ensures hydrodynamically smooth walls [43]. The two idealized models (with a wall thickness of 0.6 mm) at 50 μm layers resulted in even higher enhanced surface finish and clarity compared to patient cases, contributing to improved imaging quality. Steps 4 and 7 in the flowchart (Fig. 1) display samples of printed models. Our high-resolution imaging did not show any measurable wall motion or deformation in any of the models.

### Flow Loop

**Fig. 3:**
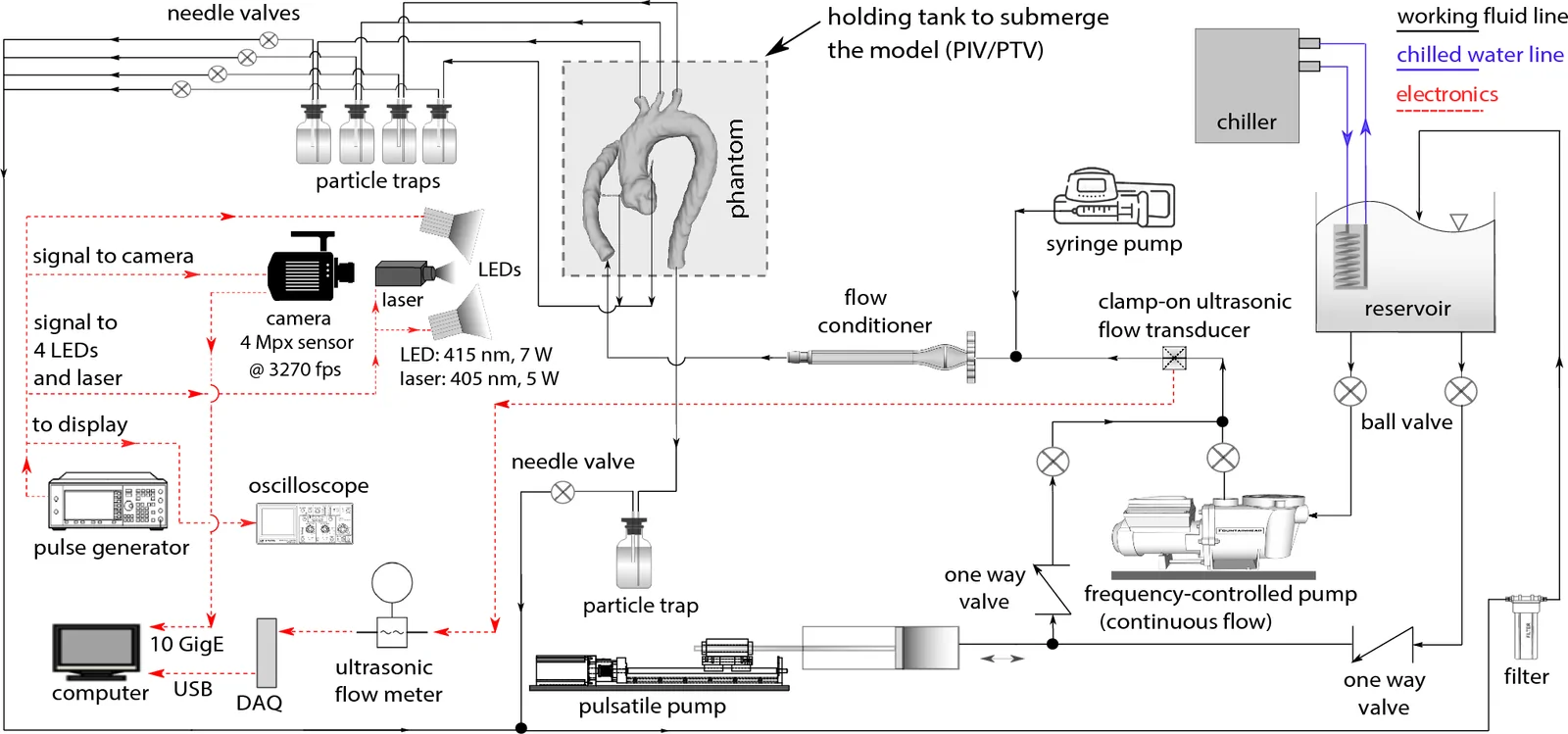
A universal experimental setup developed for PIV/PTV experiments. In this study, only the continuous-flow pump was used. The schematic is not to scale

The 3D-printed aortic models are submerged in a water tank and placed into a flow loop, where steady, pre-specified flow rates, matching the operating conditions of blood in LVAD systems, are imposed. Figure 3 illustrates a schematic diagram of the experimental setup designed to offer instrumentation for PIV/PTV studies applicable to any fabricated model.

The rig is equipped with both continuous and pulsatile flow-driven pumps. Depending on the flow conditions being investigated, each pump can be utilized to impose the desired flow into the model. However, in this particular study, only the variable-frequency continuous pump was used. The flow loop can use water or a mixture of water and glycerol. For practical purposes in this study, water was used as the working fluid. As the inertial particles are sensitive to the working fluid density, to ensure being neutrally buoyant in water, experiments are performed at a temperature of ∼18 °C with a kinematic viscosity of $\nu \approx 1.053 \times 10^{-6}$ m$^2$/s and a density of $\rho \approx$ 998.6 kg/m$^3$. For this purpose, an 800-watt CW-5000 Industrial Water Chiller circulated coolant through a copper coil in the holding reservoir to keep the working fluid temperature constant.

Two steady-state flow rates equivalent to 5 L/min and 9 L/min of blood flow are used. For this purpose, the Reynolds number, $Re_D = U_{bulk}D/\nu$, as the governing dimensionless parameter, is matched between the *in vitro* measurements and the physiological conditions, as if the working fluid were blood; where $U_{bulk}$ is the bulk velocity (cross-sectional average) at the cannula inflow with an inner diameter of D=16 mm, and ν is the fluid kinematic viscosity that is commonly assumed as $3.77 \times 10^{-6}$ m$^2$/s for blood.

Flow rate settings are inspired by the Abbott HeartMate 3 LVAD [44]. A flow rate of 5 L/min represents common physiological cardiac output and is close to the nominal HeartMate 3 setting. A flow rate of 9 L/min approximates peak cardiac output and is near the device’s maximum setting. We selected this value as a high-flow bounding condition to ensure robust measurement of embolic transport across all cases. Although higher than typical clinical operation, prior studies indicate that flow rates up to 10 L/min are physiologically attainable under high-demand conditions, providing insight into upper-bound particle-transport behavior in the LVAD-aortic system [45].

Precision fluorescent green polyethylene beads from Cospheric LLC with a nominal diameter of 500, 850, and 1200 μm and a density of 1000 ± 10 kg/m$^3$ are used as embolic particles. The chosen particle density is consistent with experimental measurements of real blood clots reported by, e.g., [46], which indicate near-neutral buoyancy. A precision syringe pump (Chemyx Inc. Fusion 200) is used to inject the particles suspended in a water solution, replicating emboli at the two aforementioned clinically relevant flow rates.

The embolic particle volume fraction in the injection solution is $\phi = N V_p/V_{tot}$ where *N* is the number of particles in the solution of volume ${V_{tot}}$. In our experiments, this corresponds to $\phi \approx$ 0.02 for lower and $\phi \approx$ 0.04 for higher flow rates. These values of particle concentration in the injected solution correspond to an overall volume fraction of $\phi =1.5 \times 10^{-4}$ within all aortic models at both flow rates. Based on the classification map proposed by [47], the particle-flow interaction can be considered as a two-way coupling flow for this volume fraction range. Particles are injected into the flow through a designed injector with an inner diameter of 2.5 mm. The injector, positioned upstream of the flow-conditioning section, releases particles from the center of the inlet cross-section at controlled rates. The flow conditioner ensures a fully developed, symmetric inflow at the model entrance, minimizing perturbations, swirl, and any minor disturbances introduced by particle injection. The inflow has been fully characterized as detailed in subsection Boundary conditions.

The major experimental parameters, including flow conditions and other related parameters utilized in this *in vitro* study, are given in Table 1. Two steady-state flow rates of $Q_1 \approx$ 1397 mL/min and $Q_2 \approx$ 2497 mL/min are considered, corresponding to the blood flow of 5 and 9 L/min, respectively. The level of particle inertia is measured by the Stokes number $Stk=\tau _p/\tau _f$, where $\tau _p=\rho _p d_p^2/(18\mu )$ is the particle relaxation time and $\tau _f=D/U_{bulk}$ is the characteristic time scale of the fluid flow. The particle diameter is $d_p$, the particle density is $\rho _p$, and μ represents the working fluid dynamic viscosity.

**Table 1:** A summary of the experimental conditions utilized in this study

Modality	$d_p$ [μm]	*Q* [mL/min]	$U_{bulk}$ [mm/sec]	$Re_D$	$\tau _p$ [sec]	$\tau _f$ [sec]	*Stk*	ϕ
PIV	150$^1$	1397$^5$	116	1757	0.0009	0.138	0.0072	NA
PIV	150	2497$^6$	207	3163	0.0009	0.077	0.013	NA
PTV	500$^2$	1397	116	1757	0.0132	0.138	0.096	0.00015
PTV	500	2497	207	3163	0.0132	0.077	0.170	0.00015
PTV	850$^3$	1397	116	1757	0.038	0.138	0.276	0.00015
PTV	850	2497	207	3163	0.038	0.077	0.494	0.00015
PTV	1200$^4$	1397	116	1757	0.076	0.138	0.551	0.00015
PTV	1200	2497	207	3163	0.076	0.077	0.984	0.00015

The working fluid is water at* T*=18 $^o$C (ρ = 998.6 kg/m$^3$ and μ=1.052 $\times 10^{-3}$ Pa.s). The particle diameter is $d_p$, and the particle density is $\rho _p$ = 1000 ±10 $\mathrm {kg/m^3}$. $Re_D$ is the inflow Reynolds number based on the bulk velocity, $U_{bulk}$, at the cannula inlet with an inner diameter of *D*=16 mm. ϕ shows the particle volume fraction within the aortic models. $Stk=\tau _p/\tau _f$ is the Stokes number, where $\tau _p$ and $\tau _f$ are the particle relaxation time and flow characteristic time scale, respectively. All particle sizes are neutrally buoyant in the flow, thus the density ratio is $\rho _p/\rho \approx 1$

$^1$ Size range: 125–150 μm, $^2$ size range: 425–500 μm, $^3$ size range: 710–850 μm, $^4$ size range: 1000-1200 μm. For calculations, the nominal diameters are used. $^{5, 6}$ The two reported flow rates are the average of all measurements across various cases and are equivalent to 5 L/min and 9 L/min blood flow rates, respectively

### Boundary Conditions

The boundary conditions applied to the inflow and outflows of our models were carefully determined and monitored. Flow was first routed through an in-house flow conditioner. A divergent-convergent flow conditioner was designed, 3D-printed, and installed upstream of the phantom to deliver a symmetric, fully developed inflow while attenuating upstream disturbances. The flow is first decelerated through a smooth expansion and lattice insert that breaks down any swirling motions and flow non-uniformities, then re-accelerated through a contraction to establish a uniform conditioned inlet. The conditioned flow was then directed into the cannula, which diverted it to the outlet vessels: the two coronaries, three arch vessels, and the descending aorta. Plastic tubing connects these outlets to separate containers for particle collection. In accordance with the agreed anatomical flow rates, the flow from cannula is divided between the outlet vessels as follows: 15% of the inflow is sent to the brachiocephalic artery (BA), 7.5% to the left common carotid artery (LCA), 7.5% to the left subclavian artery (LSA), 5% to two coronary arteries combined, and the rest (65%) to the descending aorta. The flow rate partition is based on the duplex ultrasound data reported in the previous studies [36] as used in [40]. The flow division is achieved by an iterative process of tuning the needle valves at each outlet. The real-time flow rates are measured by a precision ME-9PXL (0–10 L/min) Transonic clamp-on flow meter with an absolute accuracy of ± 10% of the reading. A TS410 Transonic tubing flow module at a 40 Hz low-pass filter provided high-fidelity flow signals.

The inflow as a critical boundary condition was experimentally characterized using a straight tube replicating the cannula geometry of the aortic models. PIV/PTV measurements, conducted with a 45$^o$ laser light sheet orientation (with respect to the pipe axis), provided a reliable benchmark for the inflow velocity and particle concentration profiles. For this purpose, a high-resolution SLA 3D-printed tube (matching the cannula inner diameter with a wall thickness of 0.3 mm) was incorporated into the flow loop, and fluorescent microspheres were introduced to analyze particle transport. Within the inlet pipe, we conducted a rigorous experimental campaign to map particle concentration, particle velocity, and turbulence intensity, among others, of which a subset of characterization is provided in Appendix A. Experiments were conducted under the same conditions as the aortic model measurements, including particle sizes, two flow rates, and environmental parameters.

### Particle Weight Measurements, Convergence, and Uncertainties

In the setup, each LVAD outlet is directed to a separate container (as shown in Fig. 3), allowing particles to be individually collected from each branch. To ensure accuracy, we first verified measurement convergence by confirming that results stabilized after injecting a specified volume of the particle-fluid injection suspension. For this purpose, we have conducted the particle injection process incrementally for one of the patient-specific cases (Patient 1) and for the idealized Type I model. Precise weight measurements were conducted using a scale with automatic internal calibration (TORBAL - AGCN120, featuring an analytical balance) that keeps the scale calibrated at all times with an accuracy of 0.1 milligrams. Based on our assessment, 160 mL of injection solution was deemed more than sufficient and was therefore used throughout the study. Further details on the convergence test are provided in Appendix B.

Given the sensitivity of our results to measurement uncertainty, we carefully quantified branchwise uncertainties for both flow rate and particle weight. For flow rate, we calculated the overall uncertainty value (*Un*), by combining the precision limit (*Pr*) and bias errors (*B*) for each outlet’s flow ratio across all experiments. The branchwise flow rate uncertainties across all branches and cases are in the range of 0.5 to 2.2 percent of the measured values, as detailed in Appendix C. In section Results, these overall flow uncertainties are shown as gray shaded bands around the corresponding mean flow-ratio values.

In conjunction with assessing flow uncertainty, we conducted an analysis to quantify uncertainties in particle weighing. Three identical repetitions of particle weight measurements were performed using the idealized model (Type I) for each particle size at the lower flow rate of $Q_1 \approx 1397$ mL/min. Subsequently, the precision error was evaluated for each case using the same method as the flow rate. By knowing the scale’s resolution, the overall uncertainties are then calculated in the range of 0.6 - 3.4 percent of the measured values, as detailed in Appendix C. In section Results, the error bars denote the overall uncertainties around the mean particle-weight ratios.

### PIV/PTV Measurements and Sub-pixel Accuracy

For PIV/PTV measurements, flow is seeded with a series of 150, 500, 850, and 1200 μm precision fluorescent green polyethylene beads with the density of $\rho _p=$1000 $\mathrm {kg/m^3}$. First, we obtained Eulerian velocity fields via planar refractive-index-matched PIV. Our smallest particles (150 μm) were used for PIV. Their Stokes numbers are approximately 0.0072 and 0.013 under low and high Reynolds number inflow conditions, $Re_D\approx$ 1757 and $Re_D\approx$ 3163, respectively, which are sufficiently small for them to behave as tracer particles in these flow regimes. Fluorescent beads introduced into the flow are illuminated by a 5 W 405 nm continuous-wave laser, generating a ∼2 mm thick light sheet at the model’s centerplane, supplemented by four Thorlabs 7 W 415 nm DC LED lights.

LED illumination was used for PTV to provide volumetric lighting and maintain particle visibility throughout the three-dimensional flow field. Although the camera recorded a 2D projection, volumetric illumination was necessary to avoid losing particles as they may have moved along the optical axis. In contrast, planar PIV employed a laser sheet to generate the illumination plane required for standard 2C-2D velocity measurements. Illuminated beads are imaged using a Zeiss Milvus 135 mm f/2 lens in conjunction with a narrow band-pass optical filter at 518 nm (Bi518-77 from MIDOPT Inc.). This ensures an effective imaging of particles at high signal-to-noise ratios. A high-speed camera (PHANTOM T1340, 12-bit 2048 × 1952 pixels @ 3270 Hz) at frame rates of 400 fps and 800 fps is employed to capture the Lagrangian motions of particles at low and high flow rates, respectively. For each experimental case, over 132,000 images (six repetitions combined) are recorded. For PIV measurements, for each case, a sequence of 10000 images was recorded at 200 Hz and 400 Hz for the low and high flow rates, respectively.

F-numbers of 4 and 11 were selected for PIV and PTV measurements, respectively, to match the illumination strategy. Using the relations provided in [48], the theoretical in-focus depth along the optical axis was estimated as ∼1.5 mm for PIV and ∼11.5 mm for PTV. For PIV, this depth of focus, given the tracer particle size and a laser-sheet thickness of ∼2.0 mm, was sufficient to capture the fluorescent particles in focus. For PTV, visual inspection revealed an apparent depth of focus of approximately 25 mm, comparable to the aortic diameter, significantly larger than the theoretical prediction. A likely cause is that larger particles (500, 850, and 1200 μm) scatter considerably more light beyond diffraction-limited diameter, which is the basis of the estimation in [48]. The differing depths of focus in PIV and PTV reflect the distinct optical requirements of the two methods. PIV uses a relatively thin laser sheet (∼2 mm) to measure Eulerian velocity fields in the model centerplane and capture the overall flow structure. In contrast, PTV relies on LED-based volumetric illumination and a much larger depth of focus (∼25 mm) to maintain particle visibility as they travel through three-dimensional flow structures. Although out-of-plane motion could not be resolved in this single-camera setup, these measurements yielded Lagrangian particle trajectories (2D projections with in-plane velocities), along with residence times and end-destinations for the large inertial particles. The stronger signal enables detection farther from the focal plane, thereby extending the effective depth of focus beyond the diffraction-limited theoretical estimate, which is based on point-like, uniformly bright emitters. The camera settings for imaging measurements are delineated in Table 2.

We obtained Lagrangian trajectories of inertial particles (500, 850, and 1200 μm) using PTV. For this purpose, MATLAB^®^ PTV code, written by researchers from Georgetown University [49], was employed to detect the particle locations and their trajectories by post-processing the captured images. Notably, during trajectory reconstruction, certain particles could not be reliably linked to their preceding or subsequent positions, resulting in interrupted tracks. To maintain data integrity, we excluded such incomplete trajectories from the statistical analyses. Repeating the experiments six times (132,000 images in total) provided a sufficient number of complete trajectories for robust statistics.

In this study, we present the PTV results for two particle sizes, 850 and 1200 μm for the two idealized models under two flow rates. For the 500 μm particles, the same volume fraction produced too many particles per frame, for which reliable particle tracking could not be performed, and thus those results are not shown. For both PIV and PTV, we first generated a “minimum-intensity image” by taking the lowest intensity value at each pixel in the sequence, then subtracted this background image from every frame. For PIV, a least-squares Gaussian fit (3 × 3 points) was used for particle peak detection, with FFT-based cross-correlation employing a 48 × 48 pixels interrogation window with 75% overlap. PIV processing was performed in PIVTEC PIVview 3.9.

For PTV, a set of parameters pertaining to particle size, intensity, and maximum displacement of particles was defined, enabling the precise localization of particle centers with sub-pixel accuracy within each frame. After reconstructing trajectories, we applied a 5-point moving average to particle coordinates to filter any imaging artifacts. This provided only modest smoothing relative to the raw tracks, sufficient to remove jitters without materially altering the trajectories. Of note, achieving accurate imaging of particles entering the two coronary vessels poses challenges, attributed to their tiny vessel dimensions and also the orientation of the model within the test section, which constrained the visibility of these arteries in the camera view. To mitigate these constraints, we present the outcomes of the particle ratio entering the three cerebral arches relative to that of the descending aorta corresponding to the flow ratio $Q_{arch}/Q_{desc}$, with the aim of preventing inadvertent inclusion of erroneous data from the coronary vessels.

**Table 2:** Imaging settings

Modality	*Q* [mL/min]	frame rate [fps]	exp. time $^1$[μsec]	exp. index $^2$	IR $^3$[μm/pixel]	f$_\#$	DOF $^4$ [mm]
PIV	1397	200	200	80000	80	4	1.5
PIV	2497	400	200	80000	80	4	1.5
PTV	1397	400	250	16000–25000 $^5$	80	11	11.5
PTV	2497	800	250	16000–25000 $^5$	80	11	11.5

$^1$ Exposure time, $^2$ Exposure index, $^3$ Image resolution, $^4$ Theoretical estimation of depth of focus. $^5$ 25000 for dp=500 μm, 20000 for dp=850 μm, and 16000 for dp=1200 μm. Exposure Index is a sensor processing control that sets the effective ISO by applying preset tone curves (and related gamma/gain) to raw images

One critical factor that affects the accuracy of PIV/PTV measurements is the refractive index mismatch between the model of study and the working fluid. In this study, two measures were taken to successfully minimize optical refraction and match the refractive indices of the model and working fluid. First, the models were 3D-printed at high resolution (50 or 100 μm layers) with ultra-thin walls of 0.6 mm for idealized models and 1.0 mm for patient-specific models, yielding smooth and transparent surfaces. Second, the phantoms were fully submerged in the working fluid within a transparent tank. We evaluated the image distortion caused by the refractive index mismatch between the fluid and the printed models in Appendix D.

## Results

### Particle Weight Ratio Statistics

To understand the particle distribution among outlet branches, particles were collected, dried, and then weighed for each outflow branch for three different sizes at two imposed flow rates, spanning the Stokes numbers from ∼0.1 to ∼1.0. We present the particle weight ratios for each outlet branch of 6 cases (four patient-specific models and two idealized models) by dividing the weight of particles entering individual branches by the total weight of particles injected at the inflow. These ratios are calculated as follows: (1) the ratio of particle weight directed to the descending aorta to the total particle weight, corresponding to the imposed flow ratio of $Q_{desc}/Q_{sum}$, (2) the ratio of particle weight directed to the three cerebral arches combined to the total particle weight, corresponding to the imposed flow ratio of $Q_{arch}/Q_{sum}$, (3) the ratio of particle weight at the two coronaries to the total weight of particles, corresponding to the imposed flow ratio of $Q_{cor}/Q_{sum}$, (4) the weight ratio of particles entered to the three arches to those directed to the descending aorta, corresponding to the imposed flow ratio of $Q_{arch}/Q_{desc}$, (5) the weight ratio of particles in the coronaries to those directed to the descending aorta, corresponding to the imposed flow ratio of $Q_{cor}/Q_{desc}$. The term $Q_{sum}$ signifies the summation of the flow rates of all individual branches, measured using the flow meter. $Q_{arch}$ represents the cumulative flow entering the three arch branches, $Q_{cor}$ denotes the total flow directed towards the two coronary arteries combined, and $Q_{desc}$ specifies the flow rate through the descending aorta. The flow split as experimental boundary conditions were detailed in subsection Boundary conditions.

**Fig. 4:**
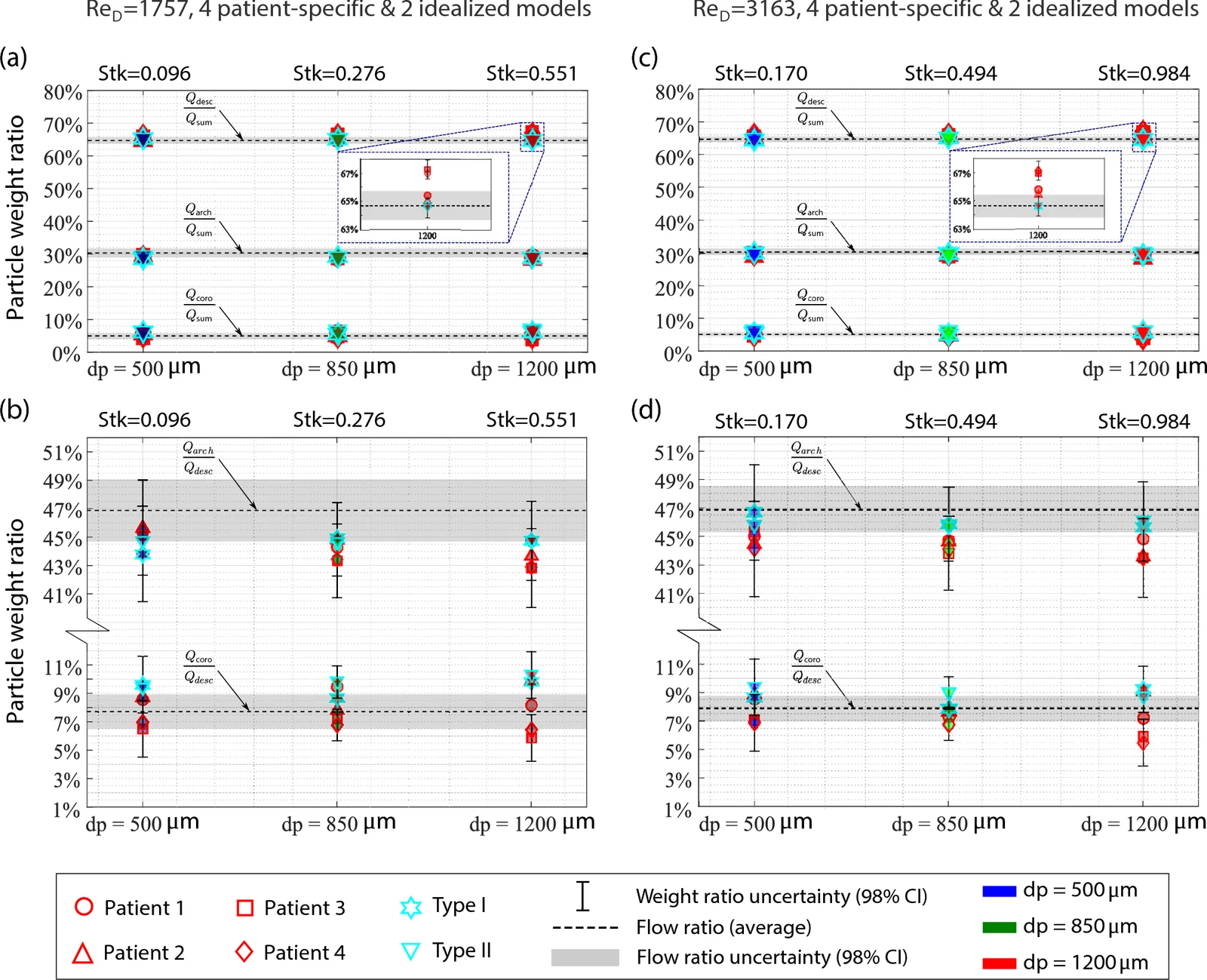
Particle distribution percentage among outlet branches for four patients (markers with red borders) and two idealized models (markers with cyan borders) with a cannula grafted at a 45-degree angle.** a** and** c** represent the particle weight ratios of descending aorta, arch vessels, and coronaries to the total particle weight for low and high Reynolds numbers of 1757 and 3163, respectively.** b** and** d** exhibit the particle weight ratios of arch and coronary branches with respect to the particle weight in the descending aorta for low and high Reynolds numbers, respectively. In each plot, the top horizontal axis provides the Stokes number corresponding to the nominal particle size on the bottom horizontal axis. In the top panels, the proportion of particles that ultimately exit via the descending aorta, supra-aortic vessels, or coronary arteries is expressed as a fraction of the total particles. In the bottom panels, the normalization is shown as a fraction with respect to particles exiting via the descending aorta. Gray bands indicate the uncertainty in the flow ratio with 98% confidence interval. Error bars show the particle weight uncertainty with 98% confidence interval and are displayed only for the minimum and maximum values for better clarity

The average values of each flow ratio, obtained from all experiments, are represented in the plots with black dashed lines for the two studied flow rates. The uncertainties, as detailed in subsection Particle weight measurements, convergence, and uncertainties, are shown as gray shaded bands around the mean values for each case. Ultimately, particle weight ratios along with their uncertainties are plotted to provide a visual representation of how particles are distributed among branches in relation to their corresponding flow division. All uncertainties encapsulate the data within a 98% confidence interval level.

Figure 4 demonstrates the particle percentage ratio taken by each outlet for all the studied cases, considering two flow rates and three particle sizes. The top horizontal axis in each plot illustrates the Stokes number, providing a correlation with the corresponding flow rate and particle size, which indicates the level of particle inertia within the flow. The bottom axis represents the particle nominal diameter. To enhance visualization, two examples of particle weight ratio (descending aorta to sum of the weight of the total particle) have been magnified, explicitly focusing on the highest Stokes number within each flow rate scenario as shown in Fig. 4(a) and Fig. 4(c).

First, we compared outcomes from four patient geometries with those obtained from two idealized aortic models to evaluate the generalizability of patient-derived findings. Although slight deviations from the flow division can be observed, particularly at higher Stokes numbers (for both low and high Reynolds numbers), we notice that for almost all cases, the error bars nearly overlap, meaning that the difference is not statistically significant, exhibiting a very good match between the patients and ideal models. This comprehensive evaluation establishes the credibility of the designed idealized aorta models in faithfully representing the complexities of patient-specific geometries.

Moreover, Fig. 4(b) and Fig. 4(d) highlight that with an increase in Stokes number, there is a slightly higher tendency of larger particles to travel toward the larger branch (i.e., the descending aorta) rather than arch vessels and coronaries. However, the overlapping error bars indicate that this effect cannot be deemed statistically significant even with a relatively high Stokes number of ∼1. The findings also reveal a consistent particle distribution trend among four patients and two ideal models, irrespective of two distinct flow rates and three particle sizes. Additionally, a notable conclusion is drawn that the overall particle distribution in arch vessels, descending aorta, and coronaries closely approximates the imposed flow division.

**Fig. 5:**
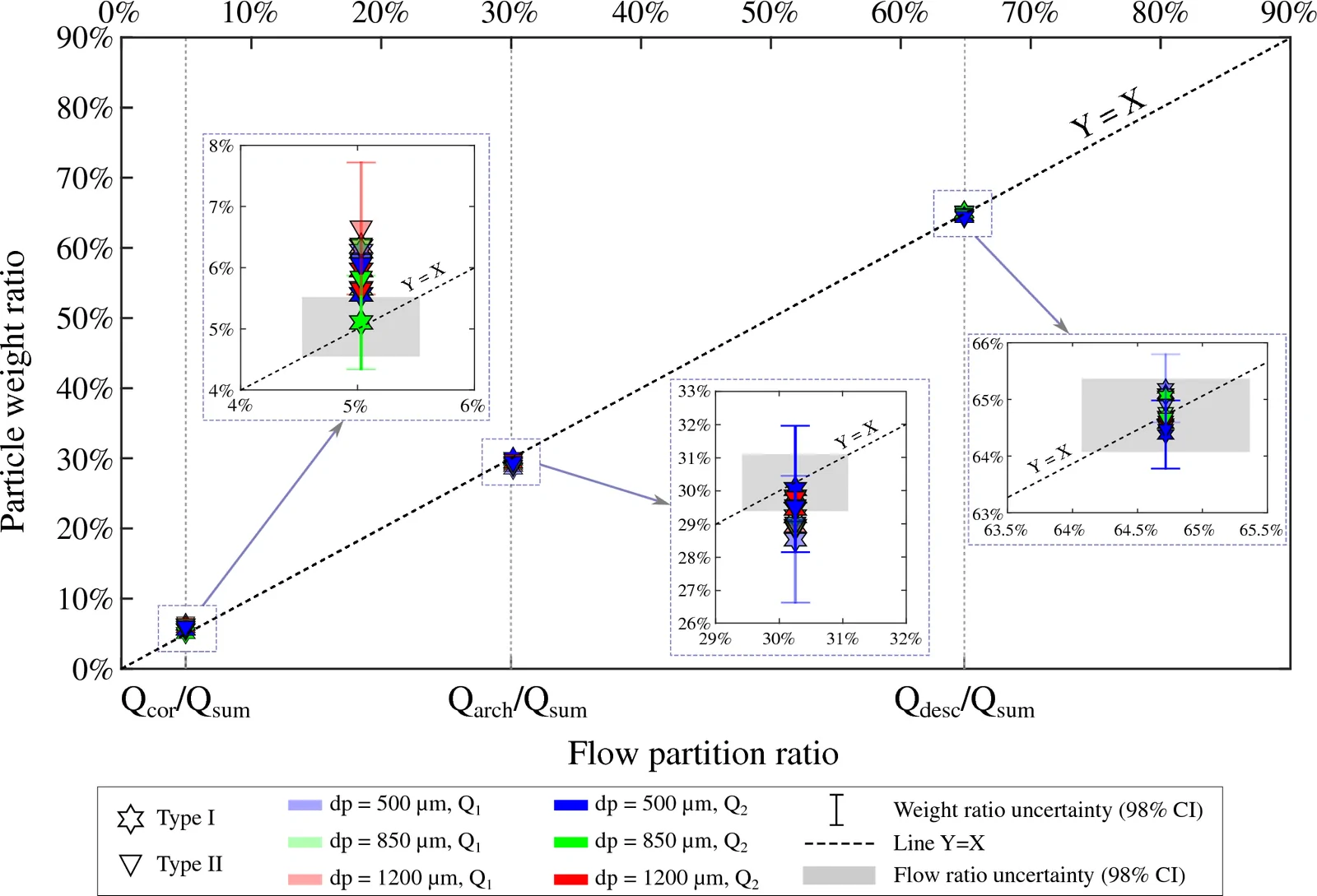
Particle weight ratio versus imposed flow partition ratio among outlet branches for Type I and Type II idealized aortic models at two flow rates for three particle sizes. The X-axis depicts the flow ratio of each outlet branch to the sum of the flow rates in all outlet branches, whereas the Y-axis is the particle weight ratio. The black dashed line (Y=X) denotes the scenario where particles follow the imposed flow split exactly. For better clarity, only the error bars of the highest and lowest data points are displayed. The gray shaded regions indicate the flow rate ratio uncertainty at the 98% confidence interval, shown bidirectionally

To evaluate the correlation between particle weight distribution in individual branches and their respective imposed flow partitions, the former is plotted against the latter in Fig. 5. The dashed equality line (Y=X) shows the scenario in which particles perfectly follow the imposed flow split. The results are provided for three particle sizes at two flow rates for the two idealized models, along with the zoomed views for better visualization. To provide an inclusive comparison, error bars at the highest and lowest points of each branch are included, allowing for a visual assessment against the flow ratio uncertainty depicted by the gray-shaded area. Figure 5 corroborates the Fig. 4 finding that, largely independent of particle size and inflow rate, particles closely follow the imposed volumetric flow split.

### PIV/PTV Results

**Fig. 6:**
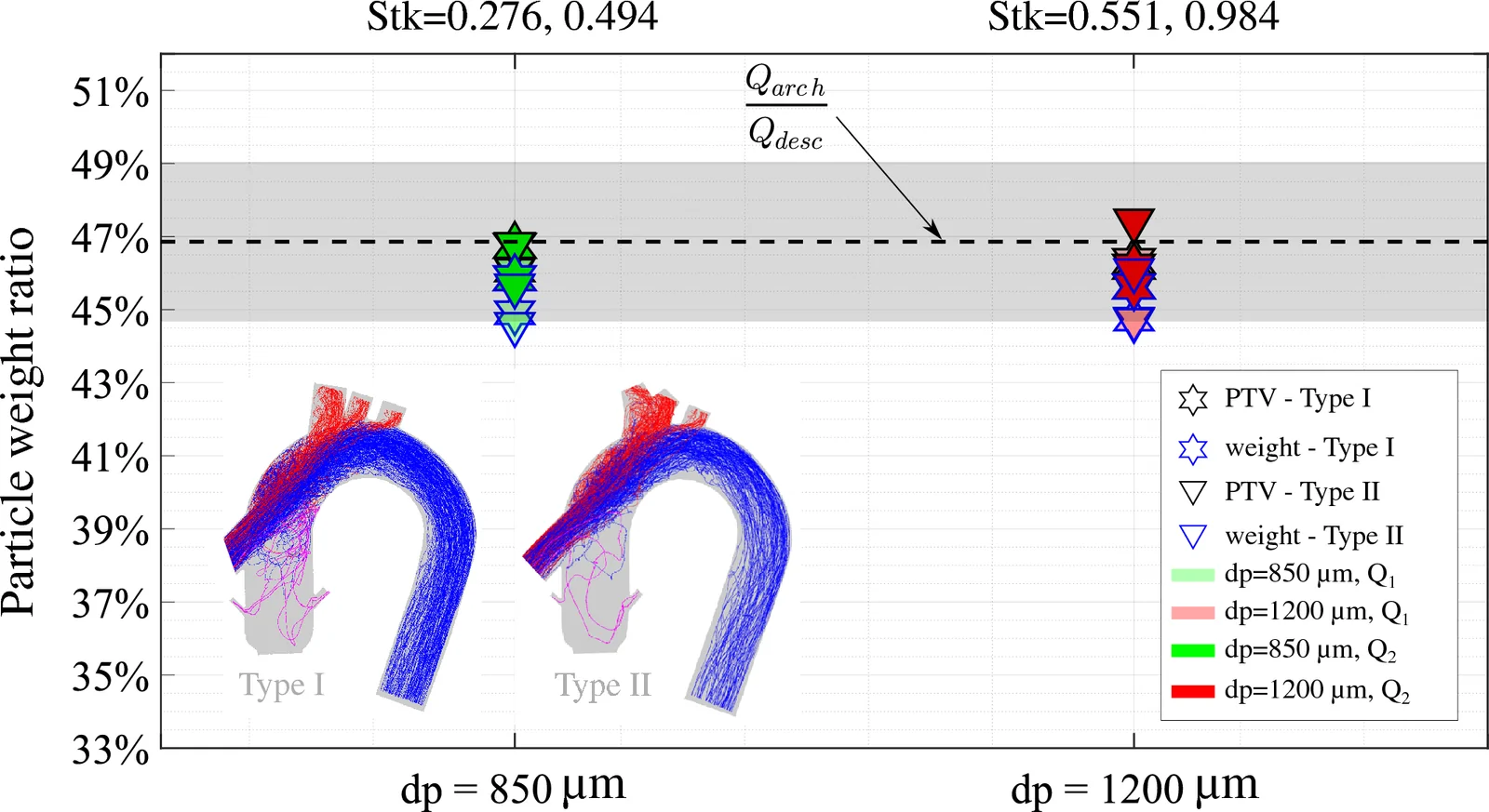
A comparison between PTV results and weight measurements, demonstrating the fraction of particles exiting the arch vessels to that of the descending aorta. The dashed line shows the flow ratio, $Q_{arch}/Q_{desc}$, with the shaded area corresponding to its measurement uncertainty with 98% confidence interval. The upper x-axis reports the Stokes numbers corresponding to the particle sizes on the lower x-axis for the low and high Reynolds number cases, respectively. A sample of particle trajectories is colored by destination outlet: blue tracks represent particles exited via the descending aorta, red trajectories depict those exited via the arch vessels, and magenta paths exhibit those that exited via the coronary arteries

Fig. 6 presents a comparison between outlet particle distributions from weight measurements and the corresponding PTV trajectory end-locations. Remarkably, our results demonstrate a close match between the two methods. The figure also illustrates a sample of Lagrangian particle trajectories, colored by the destination vessels for each LVAD idealized model. The red color represents particles going to the three arch vessels, blue corresponds to the particles traveling to the descending aorta, and the magenta color displays those directed toward the coronary arteries.

**Fig. 7:**
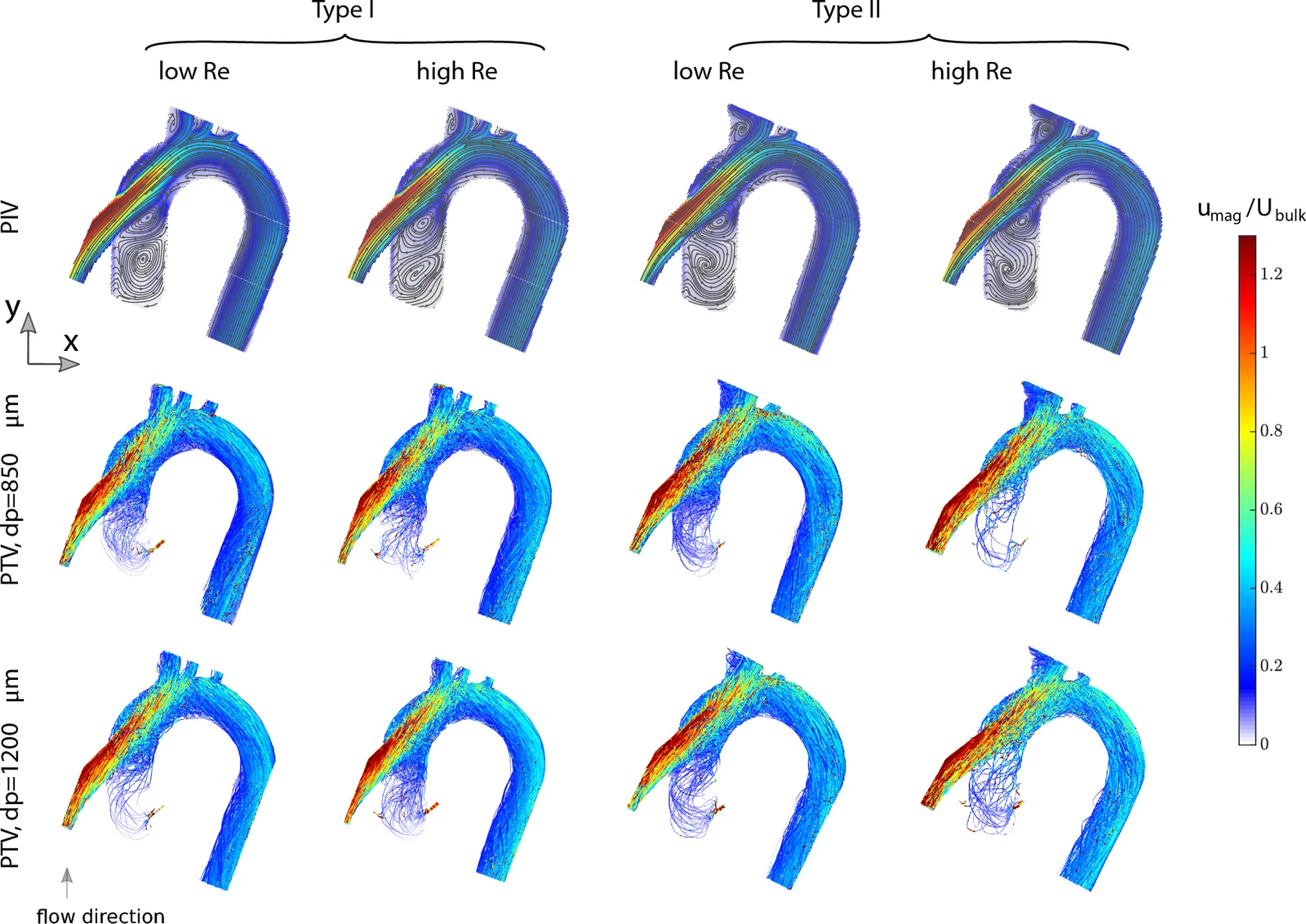
Top row: streamlines overlaid on PIV-measured velocity magnitude fields for the Type I and Type II idealized models. Middle and bottom rows: PTV particle trajectories for 850 μm and 1200 μm particles, respectively. The colorbar shows the local particle velocity magnitude normalized by the inflow bulk velocity. The Reynolds number (based on the inflow bulk velocity and the cannula diameter) is approximately 1757 and 3163 for the low and high flow rates, respectively

Furthermore, we compare the Eulerian PIV fields with the Lagrangian PTV tracks (considering two idealized models of aorta grafted with an LVAD cannula) from a global hemodynamics perspective as demonstrated in Fig. 7. The first row of the figure illustrates PIV velocity fields with overlaid streamlines. The colorbar represents the local velocity magnitude normalized by the inflow bulk velocity of each scenario. As an observation, the inflow exhibited acceleration towards the superior direction as it approached the graft location, attributable to the strong curvature of the cannula tube, which is consistent in all cases. As a result, the highest velocities do not occur in the center but are instead located closer to the outer wall of the curvature. This has also been noted in the review by [50]. The second and third rows display the Lagrangian particle trajectories for particles sized 850 and 1200 μm, respectively, colored by the particle local velocity magnitude.

Both PIV and PTV results show that the incoming flow in the cannula accelerates towards the superior direction as it approaches the graft location due to its strong curvature, which is consistent in all cases. Besides, the jetting flow entering the aorta creates several recirculation zones, evident in both types of models. At the higher Reynolds number, these zones slightly change in size or intensity but remain significantly persistent. Moreover, in this specific graft angle of 45 degrees, the inlet jet is directed towards the supra-aortic vessels, and there is no impact of the jet on the front wall. This hemodynamic analysis reveals the similarity between the two idealized models, highlighting the consistency in their hemodynamic behavior as well.

Additionally, beyond the weight-based validation of the two idealized models, we observed similar behavior between the idealized models and four patient-specific models via CFD in our prior study [42], where global hemodynamic features measured by PIV agreed with CFD in both patient-specific and idealized models.

## Discussion and Conclusions

This study highlights major gaps in LVAD particle-laden flow research: most prior work relies heavily on one-way-coupled simulations, often applies particle equations of motion beyond their valid range, and offers little to no experimental validation. These deficiencies likely drive the inconsistent embolic-risk predictions reported in the literature, and motivated us to develop a dedicated experimental framework for systematic particle-transport measurements.

We therefore developed a measurement framework that combines planar PIV and time-resolved PTV as well as outlet particle-weight measurements to quantify embolic transport in LVAD models. The campaign encompassed four LVAD patient anatomies and two idealized aortic models, each with a 45-degree anastomosis angle to the ascending aorta. Comparisons between the patient-specific and idealized models showed great agreement, supporting the use of idealized geometries as reliable surrogates for patient anatomy. The two idealized aortic models consistently produce particle distributions that are similar to those of patient-specific ones. By offering smoother walls and reduced optical distortion, they deliver higher image quality than patient models. Their transparent, low-refraction design makes them powerful testbeds for both *in vitro* and *in silico* studies, enabling controlled investigations of blood flow, thrombosis, and fluid–structure interactions. Collectively, these features provide a solid foundation for benchmarking global aortic hemodynamics and advancing cardiovascular biomechanics. Notably, we should emphasize that more localized, fine-scale hemodynamic metrics can be sensitive to patient-specific morphology; thus, the intent of the idealized aortic models is not to replace patient-specific modeling, but to provide a controlled, reproducible framework for benchmarking and mechanistic insight.

Across six LVAD configurations, we quantified branchwise embolus transport. Within the examined Stokes number range (0.1 ∼ 1.0) and particle-to-cannula diameter ratios (0.03 ∼ 0.08), particle distributions matched the imposed flow partition. Although mild deviations appear as the Stokes number increases, e.g., the clear trend observed in Fig. 4(b) and (d), the associated uncertainty bands still overlap, indicating low sensitivity to Stokes number across the full range. Our results are consistent with the previously asserted idea that particles generally tend to distribute proportionally to the fluid flow division for small (600 μm with Stk ≈ 0.262) and mid-size (1600 μm with Stk ≈ 1.863) particles, as demonstrated by [31]. They showed that only for larger particles (3200 μm with Stk ≈ 7.454), the particle distributions deviate from the flow rates, in contrast to smaller particles, which consistently adhere more closely to the flow division. This effect is crucial, particularly in situations where the vessels are not much larger than the diameter of the particles. The study by [31], however, considers an idealized bifurcation and is therefore not necessarily generalizable. Notably, our results, obtained under different geometric and flow conditions, show a similar trend. This finding, however, partly challenges the prevailing assumption that larger particles preferentially enter the larger branches. While many prior LVAD studies report particle fractions that diverge from outlet flow splits at different graft angles [34, 37, 51], our experiments show that particle ratios largely follow the imposed flow rates. Some of those earlier discrepancies likely reflect the use of much larger particles (up to 5 mm in diameter, and perhaps beyond the validity range of the particle equation of motion) or at angles other than 45 degrees, and importantly, the heavy reliance on numerical models.

The near-linear relationship between particle partitioning and flow rate partitioning may be expected to hold most closely for small, near density-matched particles with low Stokes numbers, for which advection dominates transport. For the configuration studied here, we believe particle end-destinations are largely deterministic and governed primarily by geometry. This observation is supported by consistent behavior across both patient-specific and idealized models, spanning multiple particle sizes and flow rates. In the LVAD-aorta setting examined, particles follow the imposed flow split closely for Stokes numbers up to ∼1.0. However, a linear particle-flow partition relationship is not universal and can be strongly geometry-dependent. For example, in our prior study, in a successively branching system with uniform flow-split boundary conditions, particles did not follow the imposed division even at much lower Stokes numbers (0.025 ∼ 0.25) [52], underscoring the dominant role of geometry. This example suggests that deviations from linear scaling can arise not only from inertia, but also from geometric configuration and thus the resulting flow topology.

While the present study provides valuable insights, several limitations merit note. First, we used water as the working fluid and thus neglected blood’s non-Newtonian rheology. Although certain studies have found that non-Newtonian rheology may be important [53, 54], the Newtonian assumption is typically reliable and common practice in relatively large vessels [55–58]. Second, embolic particles were modeled as rigid spheres within a certain size range, whereas clinical emboli can occasionally be larger, irregular, and deformable. These variations may alter effective inertia and, for sufficiently large particles, could influence particle transport dynamics. Third, aortic wall compliance was not modeled. Rigid walls can elevate systolic pressure, peak velocities, and pulse-wave speed, though the magnitude of these changes depends on multiple influencing factors [59]. In LVAD applications, however, a rigid-wall assumption is commonly accepted because most devices operate under steady flow. Consistent with this, we focused on steady conditions, since LVADs are commonly continuous-flow pumps, and there is little or no co-flow from the heart; under such operation, pulsatility and wall compliance play a reduced role compared with healthy aortic regimes. More broadly, introducing pulsatility would add complexity-altering unsteadiness, instabilities, vortex dynamics, and transport-through coupled effects of Reynolds, Womersley, and Strouhal numbers (e.g., [60]).

Finally, we assumed a fully closed aortic valve bypassed by the device. This simplifying assumption is the most important limitation of the present study. The presence of residual transaortic flow and whether the aortic valve (AV) opens depends on multiple factors. Depending on the native left ventricle contractility and LVAD pump speed, the aortic valve may open with every beat, intermittently, or remain persistently closed. As a reasonable ballpark, approximately 30–80% of continuous-flow LVAD patients may have a persistently closed AV at follow-up, e.g. see [61–64]. In case of aortic valve opening, weak co-flow through the valve can occur depending on patient condition and device settings, potentially modifying ascending-aorta flow and particle trajectories. Any residual ejection from the left ventricle may introduce a secondary inflow that interacts with the LVAD cannula jet. This would shift the ascending aortic flow from a steady, jet-dominated regime to a weakly-to-moderately pulsatile co-flow. Such interaction may modify shear-layer dynamics, vortex formation, flow instabilities, and aortic-root washout. For example, the patient-specific computational study by [65] showed that intermittent aortic valve opening shortens particle residence time and redistributes transport pathways relative to fully LVAD-dominant flow. Similarly, the CFD study by [37] demonstrated that embolization rates depend on thrombus origin and unsteady flow conditions, and that pulsatility modifies cerebral thromboembolism risk. Therefore, future work should incorporate flow pulsatility, residual left ventricular contribution, and LVAD operating conditions to systematically quantify thrombogenic and embolic risk across clinically relevant scenarios.

While these limitations do not diminish the study’s main conclusions, they do point to fruitful directions for future work on embolic transport in cardiovascular flows. This study examined the commonly used 45-degree anastomosis angle. In a follow-up study, we will present our work performed on systematically varying the graft angle in two idealized aortic models to identify the LVAD configuration that directs the fewest thrombi toward the cerebral vessels, providing an engineering pathway to lower ischemic-stroke risk. Clinically, as LVAD therapy continues to shift from short-term “bridge” support toward long-term “destination” therapy, the cumulative consequences of altered flow and particle transport – particularly thromboembolic risk and aortic-root/aortic-valve stasis – become increasingly important. In such cases, stroke remains a major complication during CF-LVAD support, with registry and clinical analyses documenting a burden of both ischemic and hemorrhagic events over the duration of support (e.g. [66]), as well as the risk of multifocal diffusion-restricted lesions suggestive of an “embolic shower” pattern (e.g. [67]). In principle, adjusting the anastomosis bevel, outflow cannula curvature, and proximal-distal positioning relative to the aortic valve can reduce adverse competing flows and promote more favorable washout. Optimizing these parameters may help preserve residual native output and reduce the long-term risk of stagnation-driven complications. Quantifying particle fate, as done here, provides actionable information that can inform pre- and intra-operative decision-making and guide configuration selection. This may motivate future clinical studies, leveraging patient-specific flow imaging modalities such as 4D-Flow MRI in LVAD recipients, to directly link *in vivo* flow routing with embolic distribution.

## Figures and Tables

**Fig. 8:**
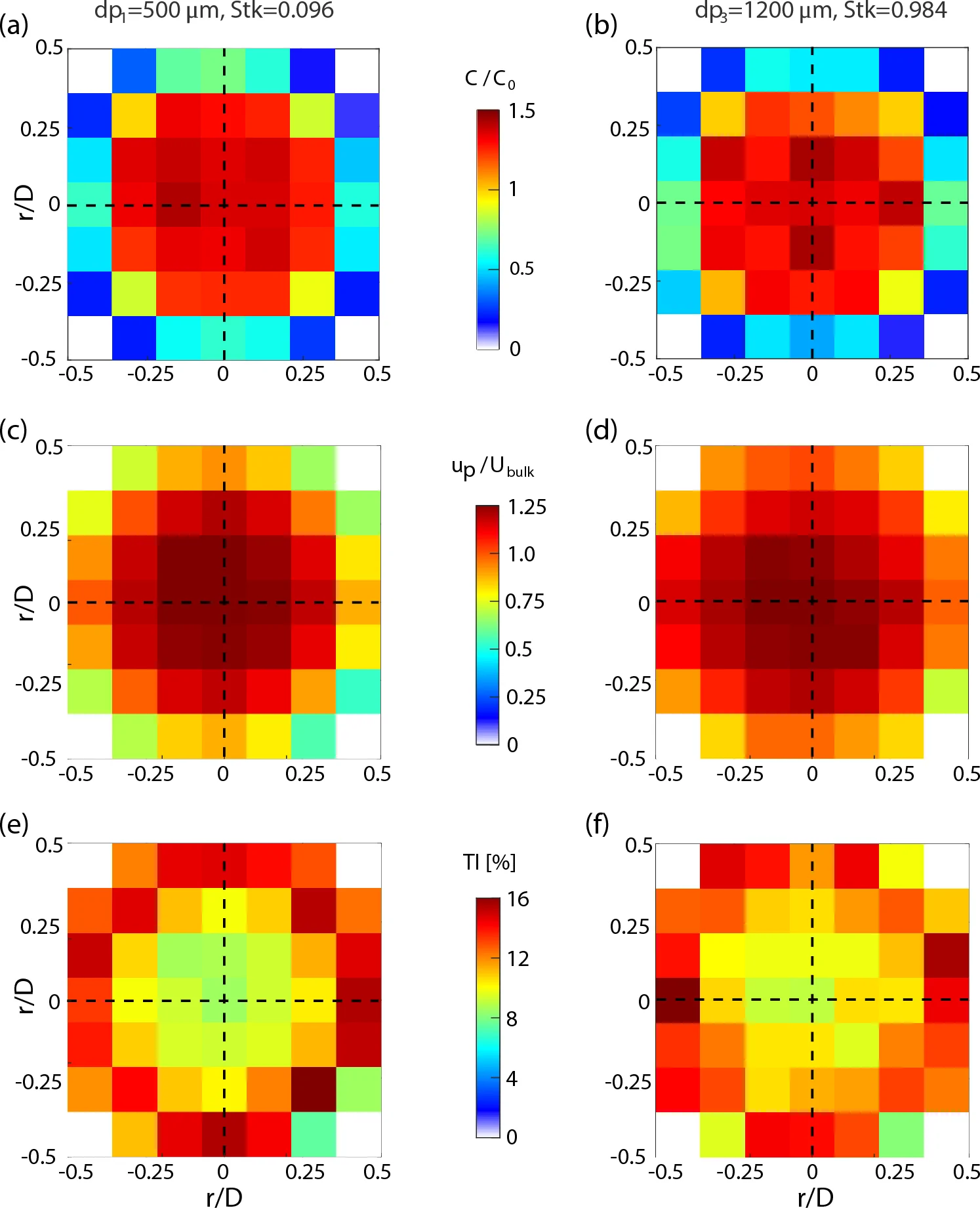
2D maps of particle statistics within the straight inflow tube. Normalized particle concentration ($C/C_0$) at **a** *Stk* = 0.096 (i.e., $dp_1$, $Q_1$) and **b** *Stk* = 0.984 (i.e., $dp_3$, $Q_2$). Particle mean axial velocity ($u_p$) normalized by the inflow bulk velocity ($U_{bulk}$) at **c** *Stk* = 0.096 and **d** *Stk* = 0.984. Particle turbulence intensity (TI) at **e** *Stk* = 0.096 and **f** *Stk* = 0.984

**Fig. 9:**
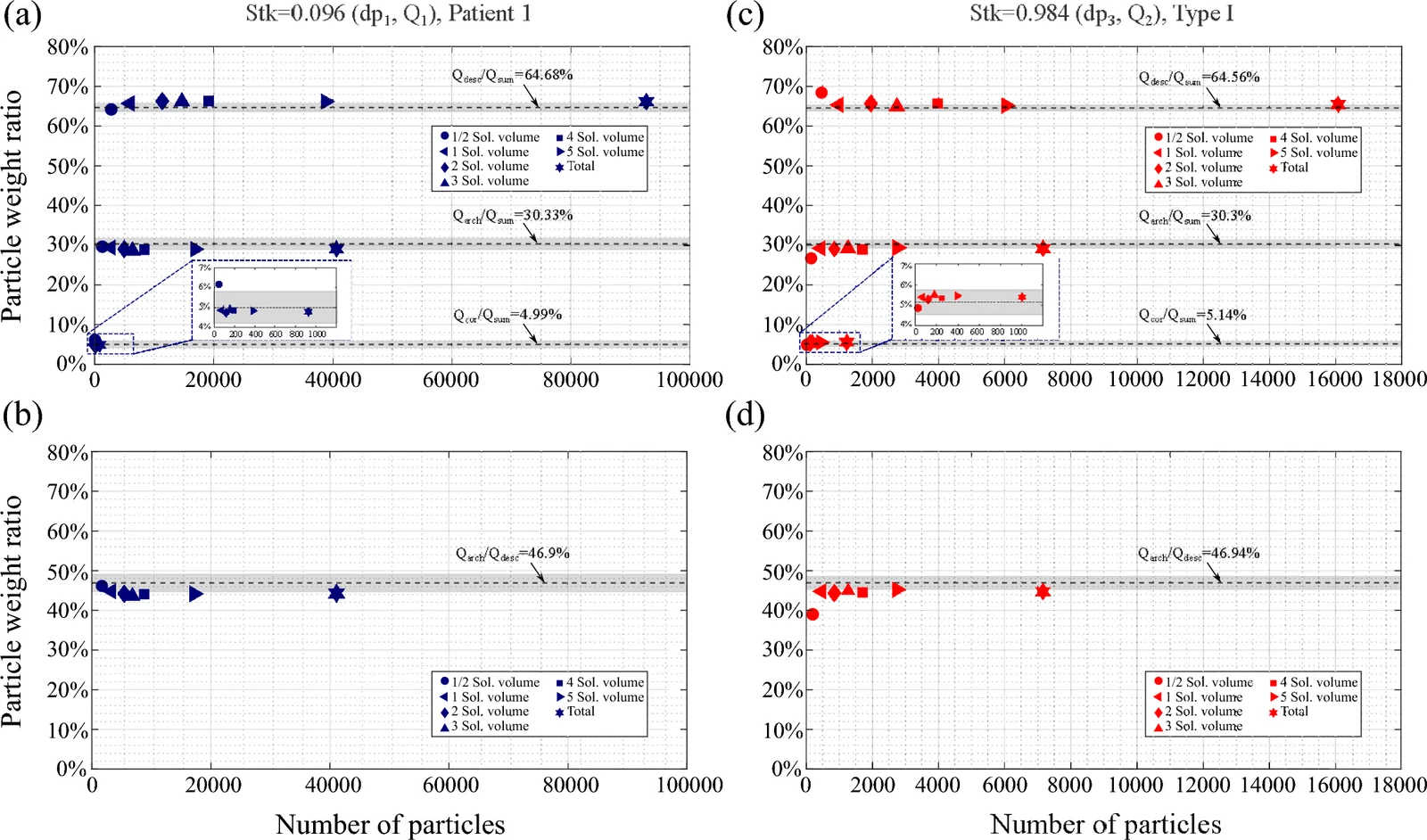
Convergence test for particle weight measurements. Panels **a** and **b** present the particle weight ratios for Patient 1 at *Stk* = 0.096, and panels **c** and **d** present the particle weight ratios for the Type I idealized model at *Stk* = 0.984. Black dashed lines mark each branch’s imposed flow rate as a fraction of total outlet flow, with gray bands denoting 98% CI. “Sol. volume” denotes the injected solution volume (one unit is ∼40 mL)

**Fig. 10:**
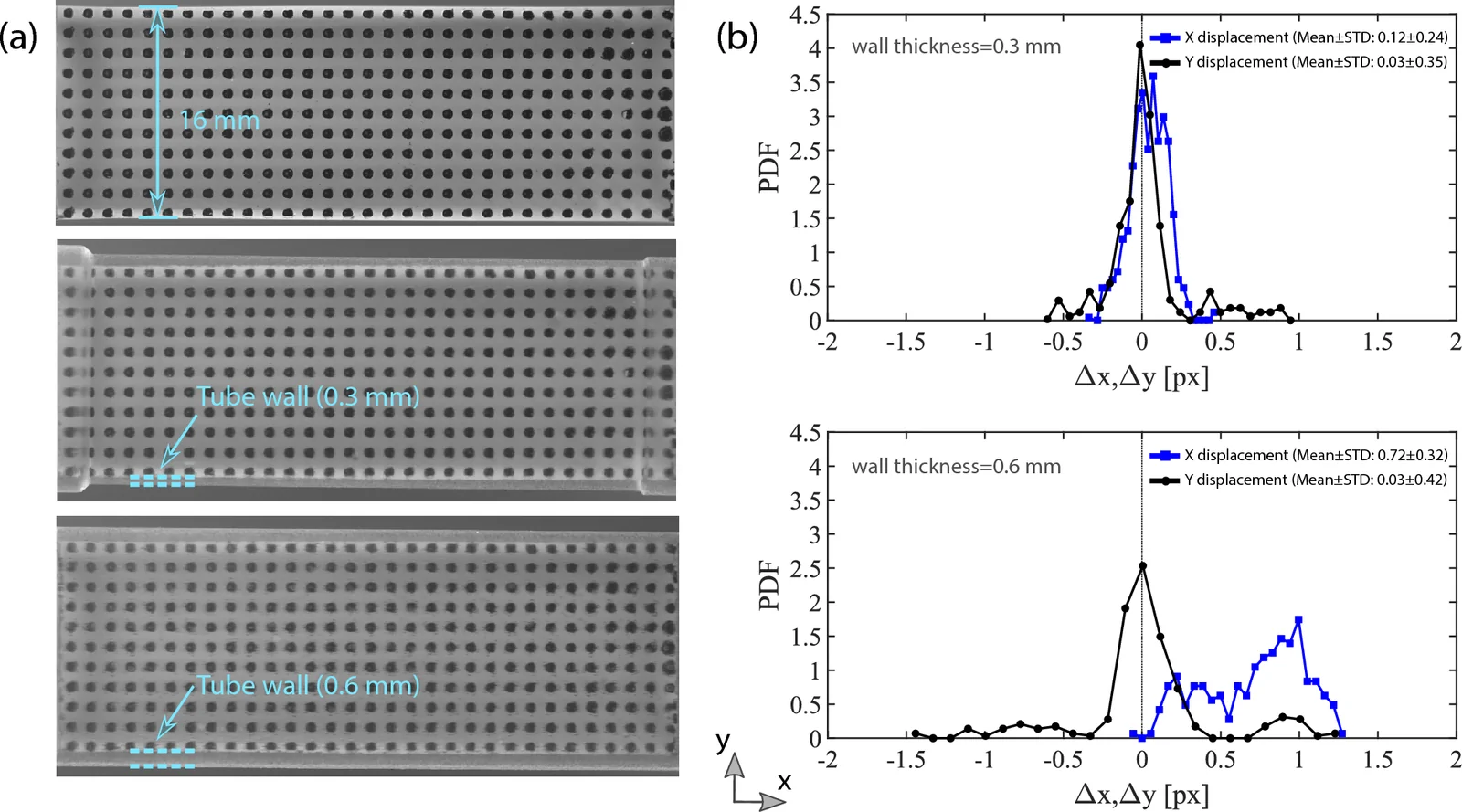
Image distortion assessment using cross-correlation of a bare grid with its view through a tube of known wall thickness.** a** Top: bare grid; middle: grid viewed through a 0.3 mm-wall tube (matching the inflow pipe wall thickness); bottom: grid viewed through a 0.6 mm-wall tube (matching the wall thickness of idealized models).** b** Probability density functions (PDFs) of pixel displacement in the x and y directions for the 0.3 mm (top) and 0.6 mm (bottom) tubes. Notably, sliding the tube over the grid can inadvertently induce a small axial shift of the grid, which may have been captured at our high imaging resolution; therefore, the measured *x*-displacement should be regarded as a conservative estimate

## Data Availability

All data generated in this study are available from the corresponding author upon request.

## Appendix A: Inflow Characterization

The laser light sheet is positioned at a 45$^o$ angle relative to the inflow tube’s longitudinal axis, creating an oval intersection with the tube section. From the camera’s perspective, however, this oval appears as a circle. To the best of our knowledge, this is the first study to apply this approach for flow characterization. Using PTV, as particles pass through the light sheet, their positions and velocity statistics are compiled into 7 bins along the radial direction, presented for two cases. Figure 8(a) and (b) show the normalized particle concentration, $C/C_0$, for two extreme Stokes numbers, 0.096 and 0.984, respectively. Here, *C* represents the local particle concentration across the pipe, and $C_0$ is the reference global particle concentration for a perfectly uniform cross-sectional distribution. From these 2D maps, we observe an increasing particle concentration from the wall to the pipe centerline. The maps also show a very good symmetry, confirming our inflow quality.

Panels (c) and (d) present the particle’s axial velocity profiles ($u_p$) in mm/sec for low and high Stokes numbers, respectively, illustrating the well-defined symmetric velocity distribution achieved. Panels (e) and (f) illustrate the particle turbulence intensity (TI) for low and high Stokes numbers, respectively, expressed as a percentage. TI was calculated from the axial and wall-normal velocity fluctuations with respect to the inflow bulk velocity. The peak values are typically observed in the near-wall region, away from the pipe center. These data serve as a critical benchmark for our study and provide the foundation for defining inflow boundary conditions. Further details on the inflow characterization can be found in [68].

## Appendix B: Convergence Test

Convergence tests were performed on Patient 1 (low Stokes number: $dp_1$, $Q_1$) and on the idealized model Type I (high Stokes number: $dp_3$, $Q_2$). We began with a half volume of particle-fluid solution (∼20 mL), measuring the particle weight in each outlet for this initial injection. Subsequently, we injected one full volume of the solution and weighed the particles in each outlet branch. The experimental procedure was continued by adding one volume to that used in the previous step. This incremental testing approach was extended until we completed the experiment by injecting a total of five units of injection solution, equivalent to a total of ∼200 mL. Each step involved measuring the weight of particles in each outlet after they had dried. Figure 9(a) and (c) present particle-weight fractions of descending, three aortic arches, and two coronaries relative to the total particle count, whereas panels (b) and (d) show the ratio of particles entering the three top arch branches relative to those entering the descending aorta.

Gray-shaded areas indicate the flow ratio uncertainty with a 98% confidence interval, and black dashed lines show the average of the corresponding flow ratio. The last data point on the plots shows the sum of all injections. As the coronary arteries receive fewer particles, their data points overlap in the main plot; therefore, a zoomed-in inset is shown for clarity. As observed, after the second or third injection, the data converged, with minimal variations in the data points, indicating that this number of injections is sufficient for convergence. However, to be on the safe side and more conservative, we performed the experiment of particle weight measurements with four injections of solution volumes (a total of ∼160 mL). Notably, particle counts were estimated from the measured branchwise particle weights by assuming nominal diameters of $dp_1$=500 μm and $dp_3$=1200 μm.

## Appendix C: Measurement Uncertainty Quantification

For a general case in which an experimental result *r* is a function of *j* measured variables $X_i$

1 $$\begin{aligned} r=r(X_1,X_2,...,X_j) \end{aligned}$$

The overall uncertainty is thus given by

2 $$\begin{aligned} (u_r)^2 = (\frac{\partial r}{\partial {X_1} } u_{X1})^2 +(\frac{\partial r}{\partial {X_2} } u_{X2})^2 +...+ (\frac{\partial r}{\partial {X_j} } u_{Xj})^2 \end{aligned}$$

where $u_{Xi}$ are the combined uncertainties in the measured variables $X_i$ [69].

In our study, we estimated the bias and precision errors for various flow ratios. Here, we illustrate the bias error calculation for one representative flow-ratio case; the same procedure was applied to all other cases. We consider the flow ratio of three top arches to the total flow sum ($Q_{arch}/Q_{sum}$), where $Q_{arch}$ itself is defined as the sum of the three flow components $Q_{BA}$, $Q_{LCA}$ and $Q_{LSA}$. Accordingly, the flow ratio can be defined as a new variable $Q=\frac{Q_{BA}+Q_{LCA}+Q_{LSA}}{Q_{sum}}$ and the bias error can be estimated for the flow ratio *Q* from Eq. 2 as follows:

3 $$\begin{aligned} & (B_Q)^2 = (\frac{\partial Q}{\partial Q_{BA}} B_{BA})^2 + ( \frac{\partial Q}{\partial Q_{LCA}} B_{LCA})^2 \nonumber \\ & + ( \frac{\partial Q}{\partial Q_{LSA}} B_{LSA})^2 + (\frac{\partial Q}{\partial Q_{sum}} B_{sum})^2 \end{aligned}$$

in which $B_{BA}$, $B_{LCA}$, $B_{LSA}$, and $B_{sum}$ represent the bias value of the corresponding branches and the sum of the outlets. These bias errors are assumed to be the same for the branchwise flow rates under the imposed $Q_1$ and $Q_2$, denoted $B_1$ and $B_2$, respectively. The values of $B_1$ and $B_2$ were determined experimentally for the ultrasonic flow meter using two different gain settings corresponding to the low and high Reynolds number conditions. Thus, the expression for $Q_1$ flow rate simplifies to

4 $$\begin{aligned} (B_Q)^2 = 3 (\frac{B_1}{Q_{sum}} )^2 + (B_1 \frac{Q_{BA}+Q_{LCA}+Q_{LSA}}{Q_{sum}})^2 (\frac{1}{Q_{sum}^2 }) \end{aligned}$$

where Eq. 4 can be applied to $Q_2$ by substituting $B_1$ with $B_2$.

To estimate the precision error, the following t-distribution criterion with 98% confidence interval was used

5 $$\begin{aligned} Pr = \frac{t ~ \sigma _Q}{\sqrt{N}} \end{aligned}$$

where *N* is the number of test repetitions and $\sigma _Q$ is the standard deviation of flow ratio *Q*. Here, *t* denotes the critical value from the t-distribution corresponding to the desired confidence level and degrees of freedom. The standard deviation of the flow ratio can be obtained using Eq. 2 as follows

6 $$\begin{aligned} & (\sigma _Q)^2 = (\frac{\partial Q}{\partial Q_{BA}} \sigma _{BA})^2 + ( \frac{\partial Q}{\partial Q_{LCA}} \sigma _{LCA})^2 \nonumber \\ & + ( \frac{\partial Q}{\partial Q_{LSA}} \sigma _{LSA})^2 + ( \frac{\partial Q}{\partial Q_{sum}} \sigma _{sum})^2 \end{aligned}$$

where $\sigma _{BA}$, $\sigma _{LCA}$, and $\sigma _{LSA}$ are the flow measurement’s standard deviation of each branch, and $\sigma _{sum}$ highlights the standard deviation value coming from the outlet’s flow summation. The flow rate of each branch was measured individually, resulting in distinct standard deviations for each. Finally, the bias and precision errors were combined to approximate the uncertainty of the flow ratio

7 $$\begin{aligned} Un = \sqrt{B_Q^2 + Pr^2} \end{aligned}$$

Table 3 shows the branchwise flow rate uncertainties, aggregated over all experiments conducted during the weight-based particle-fate measurements, underscoring the precision of our measurements.

**Table 3:** Uncertainties in flow rate ratios for the two imposed inflow conditions at the 98% confidence interval

Item [%]	*Q* [mL/min]	$Q_{desc}$/$Q_{sum}$	$Q_{arch}$/$Q_{sum}$	$Q_{cor}$/$Q_{sum}$	$Q_{arch}$/$Q_{desc}$	$Q_{cor}$/$Q_{desc}$
Precision error	1397	0.24	0.07	0.02	0.16	0.04
	2497	0.18	0.05	0.01	0.12	0.02
Bias error	1397	0.93	1.38	0.79	2.18	1.22
	2497	0.68	1.00	0.57	1.58	0.88
Uncertainty	1397	0.96	1.38	0.79	2.19	1.22
	2497	0.71	1.01	0.57	1.58	0.88

desc = descending aorta, arch = three arch vessels combined, cor = coronary arteries, sum = the total flow rate from all outlet branches

Similarly, we quantified the measurement uncertainties for the particle weight measurements. Table 4 shows the branchwise particle weight rate uncertainties, illustrating our measurement precision. Notably, uncertainty was quantified at the lower flow rate; because the weighing protocol and instrument precision are rate-independent over our test range, we expect comparable uncertainties at the higher flow rate.

**Table 4:** Uncertainties for particle weight ratios, quantified for the three particle sizes using the idealized Type I model at $Q_1=1397$ mL/min, considering 98% confidence interval level

Item [%]	$d_p$ [μm]	$W_{desc}$/$W_{sum}$	$W_{arch}$/$W_{sum}$	$W_{cor}$/$W_{sum}$	$W_{arch}$/$W_{desc}$	$W_{cor}$/$W_{desc}$
	500	0.59	1.9	1.3	3.3	1.9
Precision error	850	0.63	1.4	0.7	2.5	1.1
	1200	0.6	1.5	1.1	2.7	1.6
	500	0.15	0.13	0.13	0.2	0.19
Bias error	850	0.2	0.1	0.2	0.2	0.2
	1200	0.15	0.1	0.1	0.2	0.2
	500	0.62	1.92	1.35	3.36	1.99
Uncertainty	850	0.67	1.40	0.78	2.59	1.12
	1200	0.68	1.55	1.09	2.77	1.64

desc = descending aorta, arch = three arch vessels combined, cor = coronary arteries, sum = the total particle weight entering all outlet branches

## Appendix D: Image Distortion Assessment

To evaluate the image distortion due to the refractive index mismatch between the working fluid (water) and the model (Somos^®^WaterShed XC11122 resin), we designed and 3D-printed a grid-pattern calibration as a known reference pattern. Then, we utilized the same high-speed camera at a high imaging resolution of ∼31 μm/px to capture two image sets: bare grid and the grid viewed through a tube. This process was conducted for two tubes with an inner diameter of 16 mm (similar to the cannula diameter) and wall thicknesses of 0.3 mm and 0.6 mm; see Fig. 10(a). Cross-correlation analysis was then used to quantify pixel displacements in the *x* and *y* directions. The resulting probability density function (PDF) plots illustrating pixel movements for both cases are shown in Fig. 10(b).

The mean and standard deviation of pixel shifts are shown within the PDF plots for tubes with 0.3 mm and 0.6 mm wall thicknesses. Notably, the tube’s inner diameter matches that of the cannula tube, which exhibits one of the strongest curvatures in the aortic models. Consequently, this observed pixel displacement mainly results from the strong curvature of the cannula. This effect is expected to be much less pronounced within the more gently curved aorta. While these sub-pixel shifts are unlikely to affect particle trajectories, they may cause a slight bias in velocity estimates. Further details on the imaging can be found in [68].
